# Breast cancer hypoxia in relation to prognosis and benefit from radiotherapy after breast-conserving surgery in a large, randomised trial with long-term follow-up

**DOI:** 10.1038/s41416-021-01630-4

**Published:** 2022-02-09

**Authors:** Julia Tutzauer, Martin Sjöström, Erik Holmberg, Per Karlsson, Fredrika Killander, L. M. Fredrik Leeb-Lundberg, Per Malmström, Emma Niméus, Mårten Fernö, Annika Jögi

**Affiliations:** 1 Department of Experimental Medical Science, Lund University, Lund, Sweden; 2 Division of Oncology, Department of Clinical Sciences Lund, Lund University, Lund, Sweden; 3 Department of Oncology, Institute of Clinical Sciences, Sahlgrenska Academy, University of Gothenburg, Gothenburg, Sweden; 4 Department of Haematology, Oncology and Radiation Physics, Skåne University Hospital, Lund, Sweden; 5 Division of Surgery, Department of Clinical Sciences Lund, Lund University, Lund, Sweden; 6 Department of Surgery Malmö, Skåne University Hospital, Malmö, Sweden; 7 Translational Cancer Research, Department of Laboratory Medicine, Lund University Cancer Center at Medicon Village, Lund University, Lund, Sweden; 8 Skåne University Hospital, Malmö, Sweden

**Keywords:** Outcomes research, Breast cancer

## Abstract

**Background:**

Breast-conserving surgery followed by radiotherapy is part of standard treatment for early-stage breast cancer. Hypoxia is common in cancer and may affect the benefit of radiotherapy. Cells adapt to hypoxic stress largely via the transcriptional activity of hypoxia-inducible factor (HIF)-1α. Here, we aim to determine whether tumour HIF-1α-positivity and hypoxic gene-expression signatures associated with the benefit of radiotherapy, and outcome.

**Methods:**

Tumour HIF-1α-status and expression of hypoxic gene signatures were retrospectively analysed in a clinical trial where 1178 women with primary T1-2N0M0 breast cancer were randomised to receive postoperative radiotherapy or not and followed 15 years for recurrence and 20 years for breast cancer death.

**Results:**

The benefit from radiotherapy was similar in patients with HIF-1α-positive and -negative primary tumours. Both ipsilateral and any breast cancer recurrence were more frequent in women with HIF-1α-positive primary tumours (hazard ratio, HR_0–5 yrs_1.9 [1.3–2.9], *p* = 0.003 and HR_0–5 yrs_ = 2.0 [1.5–2.8], *p* < 0.0001). Tumour HIF-1α-positivity is also associated with increased breast cancer death (HR_0–10 years_ 1.9 [1.2–2.9], *p* = 0.004). Ten of the 11 investigated hypoxic gene signatures correlated positively to HIF-1α-positivity, and 5 to increased rate/risk of recurrence.

**Conclusions:**

The benefit of postoperative radiotherapy persisted in patients with hypoxic primary tumours. Patients with hypoxic primary breast tumours had an increased risk of recurrence and breast cancer death.

## Background

Breast cancer is the most common malignancy affecting women. Today, breast-conserving surgery followed by radiotherapy (RT) to the affected breast is part of the standard treatment for early-stage breast cancer. Systemic adjuvant therapy is selected based on patient and tumour characteristics and aims to target micrometastatic disease. About 80% of primary breast tumours express oestrogen receptor (ER) and are eligible for endocrine treatment [1].

RT after breast-conserving surgery considerably decreases the risk for ipsilateral breast tumour recurrence (IBTR), and to a minor extent also distant recurrence, and breast cancer death (BCD) [2, 3]. However, RT also confers side-effects [4–6], underscoring the importance of identifying potential patient-groups that do or do not benefit from RT. A number of factors that influence the therapeutic effect of RT have been identified in experimental systems, as well as in clinical materials [7]. The availability or shortage of oxygen was early identified as a major influencer of the outcome of RT [8, 9].

Oxygen levels are lower than those required to maintain normal metabolism and function in tissue, i.e., hypoxia, frequently occur in tumours, including breast cancer. Hypoxic adaptation at the cellular level is primarily controlled by the hypoxia-inducible transcription factors, HIF-1α and HIF-2α. Both are mainly regulated at the protein level and in response to hypoxia, the HIF alpha-subunits accumulate and become activated [10–13]. Tumour hypoxia contributes to tumour progression and therapy resistance, including RT-resistance [14], in direct as well as indirect ways [9]. Oxygen is required to make radiation-induced DNA-damage permanent, i.e. the oxygen enhancement effect. The hypoxic response, conveyed by HIF-induced gene expression, leads to altered metabolism, increased expression of growth factors, proliferation, and expression of cytokines [15]. In breast cancer, HIF-1α protein is a marker of poor prognosis and disease progression [16–18]. Upon reoxygenation the half-life of HIF-1α is in the minute range, creating a need for surrogate markers of hypoxia, such as more robust proteins induced by hypoxia, e.g. CAIX or hypoxic gene-expression signatures [19–21].

Here, we primarily aimed to test whether the hypoxia-marker HIF-1α affects the patient benefit of RT in a large population-based cohort with long follow-up of patients randomised to receive post-surgery RT or not after breast-conserving surgery. A second aim was to investigate whether tumour hypoxia and HIF-1α accumulation are associated with IBTR. Finally, we aimed to study whether hypoxic gene-expression signatures could complement or even replace HIF-1α protein detection as a prognostic or predictive marker.

## Methods

### Patients and study design

**Fig. 1:**
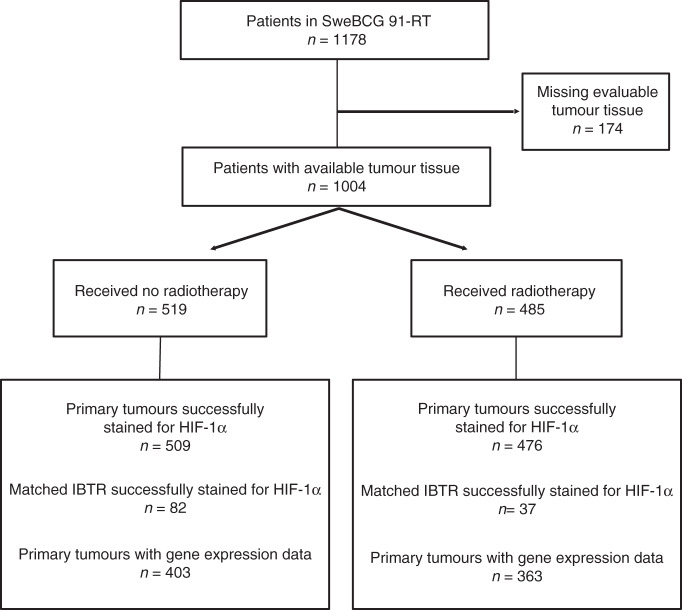
Study design. Diagram of inclusion and exclusion to the study according to Remark criteria.

Patients were from the Swedish breast cancer group trial, SweBCG91-RT, and study details are found in the previous publications [4, 5, 22–24]. Briefly, breast cancer patients with lymph node-negative (N0), stage I and IIA tumours were randomised to whole-breast RT (tangential opposed fields of 4–6 MV photons, 48–54 Gy in 24–27 fractions to the remaining breast parenchyma) or no RT after breast-conserving surgery from 1991 to 1997. Administration of systemic adjuvant treatment was according to regional guidelines of the time; 6% of patients had endocrine treatment only, 1% chemotherapy only, and 1% combined endocrine treatment and chemotherapy. The median follow-up times for event-free patients were 15.2 years (IBTR), 15.2 years (any breast cancer recurrence), 20.1 years (BCD) for the indicated endpoint. A flow diagram of the SweBCG91-RT trial is shown in Fig. 1.

### TMA construction

Tumour tissue was collected from formalin-fixed, paraffin-embedded blocks of the primary tumours from 1004 of the original 1178 randomised patients. The material includes 140 surgically treated IBTRs from patients with a primary tumour available in the TMA for matching. TMA construction was in a semi-automated TMA arrayer (Pathology Devices, Westminster, MD) by extraction of two 1.0-mm cylinders from representative tissue from each tumour block.

### Immunohistochemistry (IHC) and evaluation of markers

IHC staining, evaluation and assessment for ER, progesterone receptor (PgR), human epidermal growth factor receptor 2 (HER2) and Ki-67 were previously performed [24]. Tumours with 1% or more positive nuclei were considered ER- and PgR-positive, respectively. For dividing tumours between luminal A and B subtypes, a 20% cut-off for PgR was used. HER2 was scored with IHC as 0, 1+, 2+ or 3+ and with silver in situ hybridisation and considered positive if 3+ and/or amplified. Ki-67 scoring was according to guidelines [25], the cut-off was 10% positive cells resulting in 27% of tumours being Ki-67 high [24]. Histologic grade was previously evaluated as described by Elston and Ellis [26]. HIF-1α IHC was performed as previously described [17]. Briefly, IHC was performed on 4 μm sections of formalin-fixed paraffin-embedded sections (Autostainer Plus, Dako) according to the manufacturer’s protocol. A monoclonal antibody recognising HIF-1α (BD610959, Becton Dickinson) diluted 1:50 was employed. Two experienced evaluators blinded to patient treatment, outcome, and tumour characteristics (Kristina Lövgren and Annika Jögi) independently assessed IHC staining for HIF-1α. Each TMA-core was semi-quantitatively scored for IHC-staining intensity, 0 (negative), 1 (weak), 2 (moderate) and 3 (intense) and quantitatively scored for proportion positive cancer cells. Proportion score 0 represented less than 1% positive cells, 1: 1–10%, 2: 11–50%, and 3: 51–100%. Based on IHC intensity and proportion of positive cells each tumour sample was grouped as negative (less than 1% positive cells or 1–9% cells with intensity ≤1, Supplemental Fig. 1 A), low (1–9% of cells with intensity ≥2 or ≥10% of cells with intensity 1, Supplemental Fig. 1B) or high (≥10% of cells with intensity ≥2, Supplemental Fig. 1C). In case of discrepant staining between the two cores from the same tumour, the highest score was used. Cases (13%) with differing results between the viewers were re-evaluated in consensus. Only 60 tumours (6%) fell into the low-category whereas 227 (23%) fell into the high-category (Table 1). Due to this skew distribution, the samples of the two positive categories were merged into one HIF-1α positive group, as previously described [17]. Although not postulated in the evaluation criteria, all positive cancer cells had nuclear HIF-1α IHC-signal and very few, in addition, had cytoplasmic staining, as previously demonstrated [17]. We recently published positive and negative controls for HIF-1α immunostaining on cell lines and a similar breast cancer TMA-material of patients with contralateral tumours [17].

**Table 1:** Patient and tumour characteristics in 985 T1-2N0M0 breast cancer patients randomised to postoperative RT or no RT after breast-conserving surgery, stained for HIF-1α.

	Total	HIF-1α immunoreactivity			*p*
		Negative (%)	Low (%)	High (%)	
	985	698 (70.9)	60 (6.1)	227 (23.0)	
Age (median = 60)					
≤49	192	137 (71.4)	15 (7.8)	40 (20.8)	0.80^a^
50–59	300	210 (70.0)	18 (6.0)	72 (24.0)	
60–69	375	267 (71.2)	22 (5.9)	86 (22.9)	
≥70	118	84 (71.2)	5 (4.2)	29 (24.6)	
Premenopausal	196	137 (69.9)	17 (8.7)	42 (21.4)	0.26^b^
Postmenopausal	765	541 (70.7)	43 (5.6)	181 (23.7)	
Missing	24	20	0	4	
Tumour size (median = 12 mm)					
Tumour >20 mm	841	604 (71.8)	49 (5.8)	188 (22.4)	0.37^b^
Tumour ≤20 mm	138	91 (65.9)	10 (7.3)	37 (26.8)	
Missing	6	3	1	2	
ER-negative	100	40 (40.0)	8 (8.0)	52 (52.0)	<0.0001^b^
ER-positive	858	642 (74.8)	49 (5.7)	167 (19.5)	
Missing	27	16	3	8	
PgR-negative	253	154 (60.9)	16 (6.3)	83 (32.8)	<0.0001^b^
PgR-positive	705	528 (74.9)	41 (5.8)	136 (19.3)	
Missing	27	16	3	8	
HER2-negative	889	645 (72.6)	52 (5.8)	192 (21.6)	0.001^c^
HER2-positive	64	33 (51.6)	5 (7.8)	26 (40.6)	
Missing	32	20	3	9	
Ki-67 low	714	550 (77.0)	46 (6.5)	118 (16.5)	<0.0001^2^
Ki-67 high	244	132 (54.1)	11 (4.5)	101 (41.4)	
Missing	27	16	3	8	
Histological grade 1	146	118 (80.8)	9 (6.2)	19 (13.0)	<0.0001^a^
Histological grade 2	567	420 (74.1)	34 (6.0)	113 (19.9)	
Histological grade 3	235	136 (57.9)	15 (6.4)	84 (35.7)	
Missing	37	24	2	11	
St Gallen subgroup					<0.0001^b^
Luminal A	552	431 (78.1)	34 (6.1)	87 (15.8)	
Luminal B (HER2−)	257	181 (70.4)	13 (5.1)	63 (24.5)	
HER2+	64	33 (51.6)	5 (7.8)	26 (40.6)	
Triple negative	80	33 (41.3)	5 (6.3)	42 (52.5)	
Missing	32	20	3	9	
No RT	509	349 (68.6)	34 (6.7)	126 (24.7)	0.26^b^
RT	476	349 (73.3)	26 (5.5)	101 (21.2)	
Other treatments					0.11^b^
Chemotherapy	9	7 (77.8)	0 (0)	2 (22.2)	
Endocrine	63	43 (68.3)	6 (9.5)	14 (22.2)	
Chemo + endocrine	8	2 (25.0)	1 (12.5)	5 (62.5)	
No other treatment	905	646 (71.4)	53 (5.8)	206 (22.8)	

^a^Calculated using linear regression.

^b^Calculated using chi-squared test.

^c^Calculated using Fisher’s exact test.

### Tumour subtyping

The tumours were, as previously reported [24], subtyped according to the St Gallen International Breast Cancer Conference (2013) Expert Panel [27] as luminal A-like (ER-positive, PgR-high, HER2-negative, and Ki-67 low), luminal B-like (ER-positive, PgR-low and/or Ki-67 high, and HER2-negative), HER2-positive (HER2-positive, any ER or PgR status, any Ki-67 expression), and triple negative (ER-negative, PgR-negative, HER2-negative, any Ki-67). The HER2-positive group thus included both luminal and non-luminal tumours due to group size.

### Gene-expression analysis

Gene-expression analysis of this trial material was previously described [28]. In brief, RNA was extracted from the 922 available paraffin-fixed patient tumour samples. Patient and tumour characteristics were similar in the excluded and analysed tumours. RNA was extracted and hybridised (GeneChip Human Exon 1.0 ST microarray, Thermo Fisher) in a Clinical Laboratory Improvement Amendments certified laboratory (Decipher Biosciences). Samples from 766 primary tumours passed the quality control of RNA, cDNA, and microarray analysis (Gene-expression Omnibus GSE119295). Single Channel Array Normalisation was used for gene-expression data normalisation [29].

### Scoring of hypoxia-related expression signatures from the literature

Eleven previously published hypoxia-related gene-expression signatures, here referred to as the name of the first author of the publication, were identified from the literature. The signatures Buffa*, Buffa reduced* [30], Denko [31], Elvidge [32], Hu [33], Mense [34], Sorensen [35], and Winter* [36] were selected from a review by Harris et al [20]. Signatures marked by * are related and derived from “Winter”, where in brief, genes co-expressed with classical hypoxia-driven genes were chosen and tested in clinical tumour samples, including breast cancer [36]. The signatures from Denko, Elvidge, Mense, and Sorensen were extracted from in vitro hypoxia (1% oxygen) treated human cells. The Hu signature comprises 13 genes referred to as a VEGF-profile, 8 of the genes having a hypoxia-responsive element that can bind HIF in their promoters. The Farmer signature was based on GSEA hypoxia genes in breast cancer cells with apocrine phenotype, i.e. not based on actual hypoxic exposure [37]. The signature referred to as “Yang” was the most recently published, and based on in vitro hypoxic treatment of prostate cancer cell lines and validated in several publicly available prostate and bladder cancer materials [38]. The Hallmark of Cancer hypoxia gene signature was included since it has been widely used in literature. It is based on several sources including the Winter, Elvidge, Mense gene-expression signatures mentioned above, and includes genes regulated by the von Hippel–Lindau factor and several metabolic pathways such as the glycolysis.

Individual hypoxia scores for primary tumours in SweBCG91-RT were calculated using the singscore package in R [39], or as described by the authors. Scores were considered as continuous variables or combined into a binary variable of high or low, with the 4^th^ quartile being defined as high, and quartiles 1–3 as low, thus giving a similar proportion of hypoxic tumours as detected by HIF-1α IHC (29%). Interaction tests were performed with scores as a continuous variable to avoid introduction of bias from cut-off. If any gene ID was not matched in our expression set, synonym gene names were retrieved using the R package HGNChelper [40]. If one signature gene ID corresponded to multiple synonym IDs, a search was conducted on GeneCards database of human genes [41] to select the matching synonym. Genes were excluded if an ID match was not identified.

### Statistical methods

All statistical analyses were performed with R (3.5.2). The primary endpoint was IBTR in any quadrant of the ipsilateral breast, though 90% were located in the same quadrant as the primary tumour, as first event within 5 years [22]. Other recurrences and death by any cause were competing events. Secondary endpoints were any breast cancer recurrence within 5 years, (including IBTR, regional and distant recurrence, but not contralateral breast cancer), with death by any cause without recurrence as competing event, and BCD, with death by other cause as competing event. For the descriptive, exploratory analysis of the relationship between HIF-1α in IBTR and BCD, the start point was the date of surgery for the IBTR, and the endpoint was BCD. Cumulative incidence with competing events was displayed graphically using the R package cmprisk [42] and presented with hazard ratio (HR) and 95% confidence interval calculated using cause-specific Cox proportional hazards model. The interactions between the benefit of RT and markers of hypoxia were evaluated using cause-specific Cox proportional hazards model with an interaction term. The proportional hazards assumption was checked graphically and tested with Schoenfeld residuals [43]. As seen before in this cohort [24], HRs over the full follow-up were generally non-proportional, thus we present estimations of HR in intervals (0–5 years, 5–15 years, and >15 years for IBTR and any recurrence as first event, or 0–10 years, 10–15 years, and >15 years for BCD as first event) along with the HR for the full follow-up. All HR estimations should be interpreted as an average over the studied time interval. Associations between HIF-1α and other patient and tumour characteristics were assessed using the chi-squared test or Fisher’s exact test or tested for trend using linear regression. Statistical significance was defined as *p* < 0.05, but due to the multiple hypothesis testing performed in this study, the interpretation of *p* as level of evidence for or against the null hypothesis should be careful.

## Results

### HIF-1α in primary breast tumours

Of the 1004 tumours available in the TMA, 985 were successfully stained and evaluated for HIF-1α (Fig. 1), where 698 (71%) were HIF-1α negative. Primary tumours with IHC-staining positive for HIF-1α were similarly distributed in the RT and non-RT groups (27% and 31%, respectively). Patient and tumour characteristics are described in Table 1. Tumour HIF-1α status correlated to histological grade, with a higher frequency of HIF-1α positivity among high-grade tumours (*p* < 0.0001). Furthermore, HIF-1α positivity was associated with cell proliferation in that it correlated to high Ki-67 (*p* < 0.0001). A considerably higher proportion of ER-negative tumours, compared to ER-positive tumours, were HIF-1α positive (60% vs 25%, *p* < 0.0001). Luminal A-like tumours were the largest subgroup with 552 tumours and 22% of these were HIF-1α positive, while luminal B tumours had a 30% frequency of HIF-1α positivity (76 of 257, Table 1).

### Higher risk of recurrence and BCD in patients with HIF-1α positive primary tumours

**Fig. 2:**
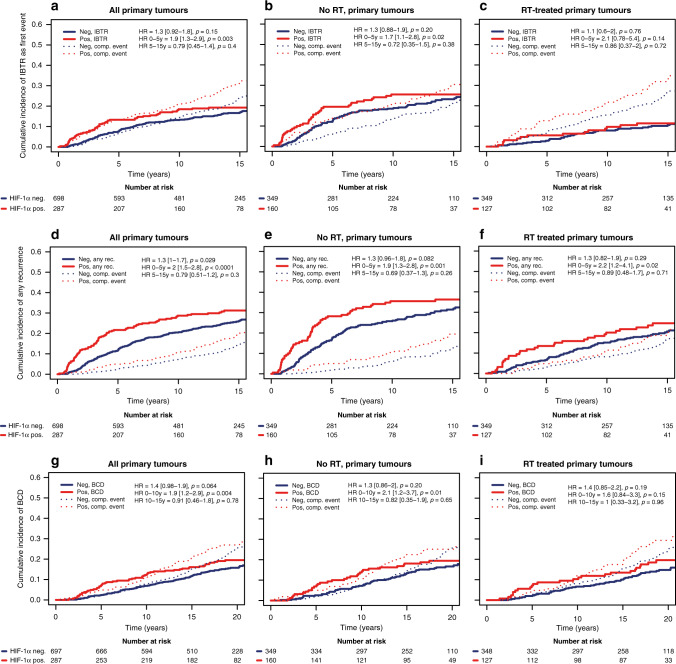
Higher risk of recurrence and BCD in patients with a HIF-1α positive primary tumour. Cumulative incidence of IBTR after surgery of the primary tumour (**a**–**c**), any recurrences (**d**–**f**), and BCD (**g**–**i**) in 985 T1-2N0M0 breast cancer patients randomised to RT (**c**, **f**, **g**) or no RT (**b**, **e**, **h**) after breast-conserving surgery in patients with HIF-1α negative (blue line) and HIF-1α positive (red line) primary tumour.

Patients with a HIF-1α positive primary tumour had an increased incidence of IBTR as a first event within 5 years compared to patients with a HIF-1α negative primary tumour both in the total patient population (HR_0–5 yrs_ = 1.9 [1.3–2.9], *p* = 0.003, Fig. 2a and Table 2) and among patients that did not receive RT (HR_0-5 yrs_ = 1.7 [1.1–2.8], *p* = 0.02, Fig. 2b and Table 2). The higher occurrence of IBTR in patients that had HIF-1α positive primary tumours was apparent in both ER-positive and -negative disease (Supplemental Fig. 2). Patients that received RT suffered less IBTR, with no difference between HIF-1α positive and negative groups (Fig. 2c). In multivariable analysis adjusted for patient age, tumour size, tumour subtype (St Gallen), and systemic adjuvant therapy, the increased risk for IBTR in the HIF-1α positive group remained an independent risk factor in the total patient population (HR_adjusted_ = 1.8 [1.1–2.8], *p* = 0.01, Table 2).

**Table 2:** Uni- and multivariable analysis of the hazard of HIF-1α (positive vs negative) in relationship to IBTR during the first 5 years after the primary tumour, any recurrences during the first 5 years after the primary tumour, and BCD during the first 10 years after the primary tumour in breast cancer patients randomised to receive RT or no RT after breast-conserving surgery.

All patients	Univariable analysis			Multivariable analysis		
	HR (95% CI)	*p*	*n* (event)	HR (95% CI)	*p*	*n* (event)
IBTR (0–5 years)						
All patients	1.9 (1.3–2.9)	0.003	985 (92)	1.8 (1.1–2.8)	0.01	948 (85)
No RT	1.7 (1.1–2.8)	0.02	509 (75)	1.6 (0.97–2.7)	0.065	492 (68)
RT	2.1 (0.78–5.4)	0.14	476 (17)	2.1 (0.78–5.8)	0.14	456 (17)
Any recurrence (0–5 years)						
All patients	2.0 (1.5–2.8)	<0.0001	985 (144)	1.7 (1.2–2.4)	0.003	948 (137)
No RT	1.9 (1.3–2.8)	0.001	509 (104)	1.6 (1–2.5)	0.03	492 (97)
RT	2.2 (1.2–4.1)	0.02	476 (40)	1.7 (0.89–3.2)	0.11	456 (40)
BCD (0–10 years)						
All patients	1.9 (1.2–2.9)	0.004	985 (83)	1.5 (0.93–2.3)	0.097	948 (82)
No RT	2.1 (1.2–3.7)	0.01	509 (47)	1.5 (0.82–2.9)	0.18	492 (46)
RT	1.6 (0.84–3.3)	0.15	476 (36)	1.3 (0.64–2.6)	0.48	456 (36)

In analyses of any recurrences in the whole patient material as well as in the non-irradiated group, HIF-1α primary tumour positivity was associated with an increase in early recurrences (HR_0–5 years_ 2, [1.5–2.8], *p* = 0.0001 and HR_0–5 years_ 1.9, [1.3–2.8], *p* = 0.001, Fig. 2d, e and Table 2), with a similar pattern in patients with ER-positive and -negative tumours (Supplemental Fig. 2). Postoperative RT led to an overall decrease in any recurrence, however, there were still a higher number of recurrences in patients with a HIF-1α positive primary tumour (Fig. 2f and Table 2).

There was a higher occurrence of BCD within 10 years of surgery in patients with HIF-1α positive primary tumour in the entire study population and in patients that did not receive RT (HR_0–10 years_ 1.9 [1.2–2.9], *p* = 0.004, and HR_0–10 years_ 2.1 [1.2–3.7], *p* = 0.01 Fig. 2g, h and Table 2), while this difference diminished after RT (Fig. 2I). In patients with ER-negative tumours, with a high frequency of HIF-1α positive primary tumours (60%), BCD within 10 years was higher compared to ER-positive tumours irrespective of primary tumour HIF-1α status (Supplemental Fig. 2e, f).

### Preserved benefit of RT in patients with HIF-1α positive primary tumours

**Fig. 3:**
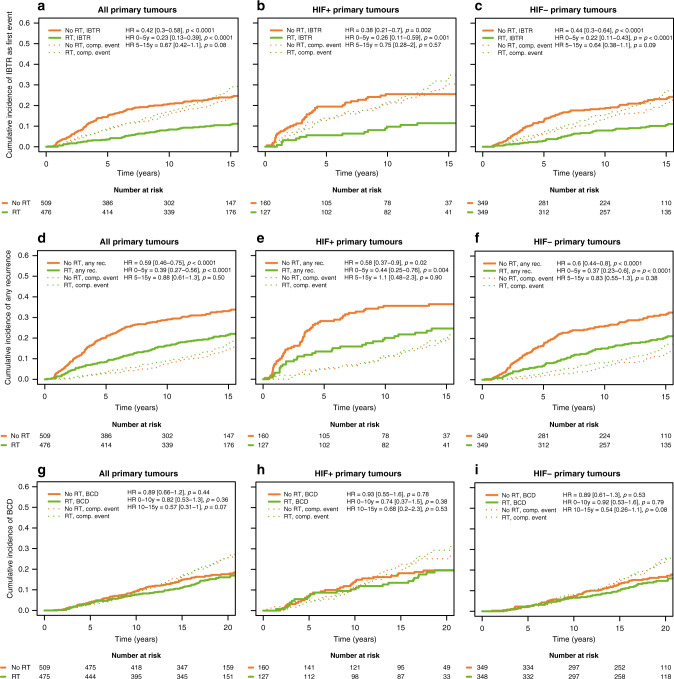
Reduction in recurrence with RT was independent of primary tumour HIF-α status. Cumulative incidence of IBTR after surgery of the primary tumour (**a**–**c**), any recurrences (**d**–**f**) as first event, and BCD (**g**–**i**) in 985 T1-2N0M0 breast cancer patients randomised to RT (green line) or no RT (orange line) after breast-conserving surgery for all primary tumours (**a**, **d**, **g**), HIF-1α positive primary tumours (**b**, **e**, **h**), and HIF-1α negative primary tumours (**c**, **f**, **i**).

Taking all primary tumours into account, patients receiving RT had a distinct reduction of IBTR within 5 years (HR_0–5years_ 0.23, [0.13–0.39], *p* < 0.0001; Fig. 3a and Table 3), and the full follow-up (HR_full FU_ 0.42, [0.3–0.58], *p* < 0.0001; Fig. 3a). Dividing the patients into those with HIF-1α negative and positive primary tumours, there was a similar reduction in IBTR with RT in the two groups (test for interaction_0–5 years_ *p* = 0.90, test for interaction_full FU_ *p* = 0.66, Fig. 3b, c and Table 3). The incidence for any recurrences within 5 years, and full follow-up, was also reduced in patients that had received RT (HR_0–5years_ 0.39, [0.27–0.56], *p* < 0.0001, and HR_full FU_ 0.59, [0.46–0.75], *p* < 0.0001; Fig. 3d and Table 3), and this effect of RT on recurrence was independent of HIF-1α status (test for interaction_0–5 years_ *p* = 0.70, test for interaction_full FU_ *p* = 0.79, Fig. 3e, f and Table 3). In the present study, there was no statistically significant effect of RT on the incidence of BCD (HR_0–10 years_ 0.82 [0.53–1.3], *p* = 0.36, Table 3), and this was unaffected by HIF-1α status (test for interaction_0–10 years_ *p* = 0.54, test for interaction_full FU_ *p* = 0.80, Fig. 3g–i, Table 3).

**Table 3:** Uni- and multivariable analysis of the benefit of RT in relationship to IBTR during the first 5 years after the primary tumour, any recurrences during the first 5 years after the primary tumour, and BCD during the first 10 years after the primary tumour in breast cancer patients randomised to receive RT or no RT after breast-conserving surgery.

	Univariable analysis			Multivariable analysis		
	HR (95% CI)	*p*	*n* (event)	HR (95% CI)	*p*	*n* (event)
IBTR (0–5 years)						
All patients	0.23 (0.13–0.39)	<0.0001	985 (92)	0.25 (0.14–0.42)	<0.0001	948 (85)
HIF-1α+	0.26 (0.12–0.59)	0.001	287 (38)	0.30 (0.12–0.69)	0.005	272 (35)
HIF-1α−	0.21 (0.11–0.43)	<0.0001	698 (54)	0.22 (0.11–0.46)	<0.0001	676 (50)
Any recurrence (0–5 years)						
All patients	0.39 (0.27–0.56)	<0.0001	985 (144)	0.42 (0.29–0.61)	<0.0001	948 (137)
HIF-1α+	0.44 (0.25–0.76)	0.003	287 (62)	0.48 (0.27–0.86)	0.01	272 (59)
HIF-1α−	0.37 (0.23–0.6)	<0.0001	698 (82)	0.37 (0.23–0.62)	<0.0001	676 (78)
BCD (0–10 years)						
All patients	0.82 (0.53–1.3)	0.36	985 (83)	0.87 (0.56–1.4)	0.54	948 (82)
HIF-1α+	0.74 (0.37–1.5)	0.38	287 (35)	0.84 (0.42–1.7)	0.62	272 (35)
HIF-1α−	0.92 (0.53–1.6)	0.79	698 (48)	0.97 (0.54–1.7)	0.91	676 (47)

### HIF-1α in IBTR and relation to outcome

**Fig. 4:**
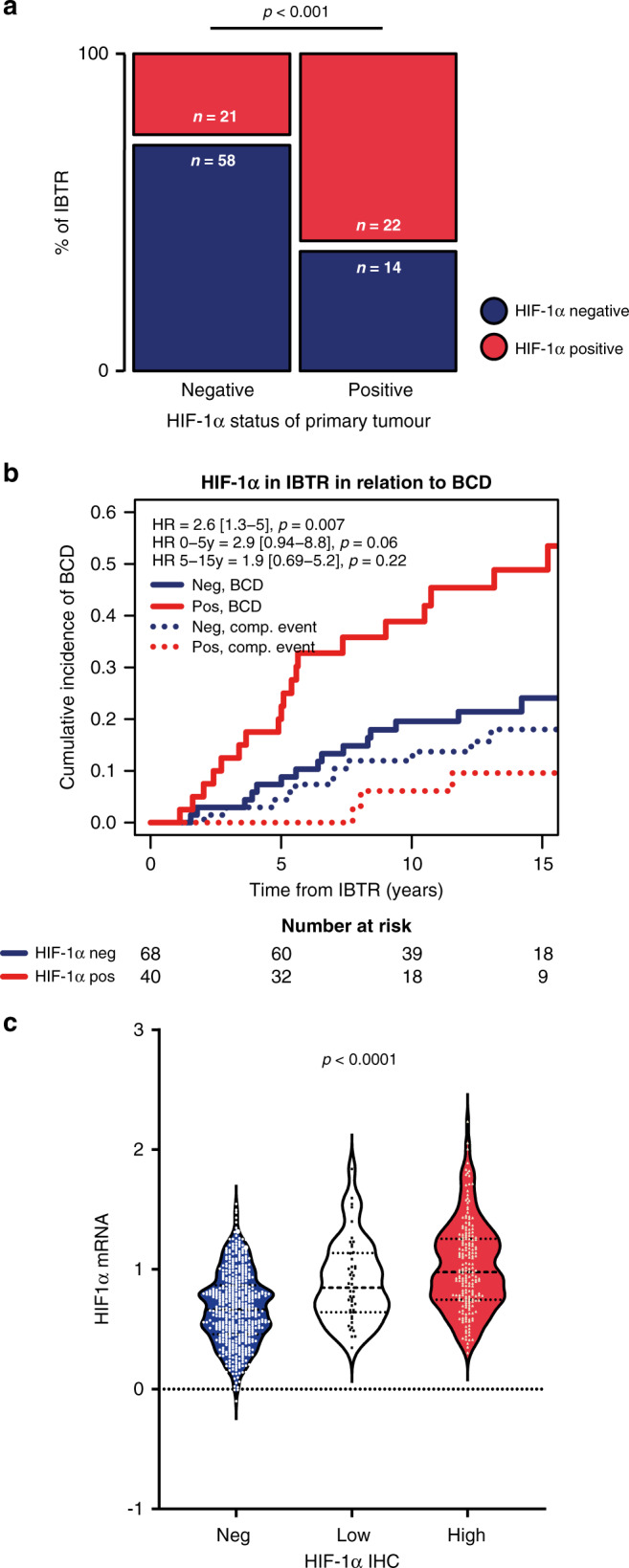
HIF-1α and IBTR. Distribution of HIF-1α positive (red boxes) and negative (blue boxes) IBTRs in relation to primary tumour status, statistically tested using the chi-squared test (**a**). Cumulative incidence of BCD in relation to HIF-1α status of the resected IBTR, with the date of surgery for the IBTR as starting point (**b**), patients with HIF-1α positive IBTR (red line) compared to those with HIF-1α negative IBTR (blue line). Time from IBTR resection. Concordance between HIF-1α IHC and *HIF-1α* mRNA levels in tumour samples, tested using Spearman’s rank test (**c**), showing HIF-1α IHC signal in three levels; negative (blue), low (white), and high (red).

For a fraction of the cohort, IHC staining of HIF-1α in matched primary and IBTR tumour material was available (*n* = 119). HIF-1α positive IBTR was more prevalent among patients that had a HIF-1α positive, compared to negative, primary tumour (61% vs 27%, *p* < 0.001: Fig. 4a). In line with this, when considering HIF-1α IHC on three levels (negative, low and high), the IBTRs most often had the same HIF-1α staining intensity as their corresponding primary tumour (65%, *n* = 75), while 21% (*n* = 24) of the IBTRs had increased intensity, and 14% (*n* = 16) had decreased HIF-1α intensity. Addressing the prognostic relevance of HIF-1α expression in IBTR, we found that HIF-1α positivity in IBTR was associated with an increased risk of BCD (HR_fullFU_2.6 [1.3–5.0], *p* = 0.007; Fig. 4b).

### Hypoxic gene-expression signatures and relation to outcome and benefit of RT treatment

**Fig. 5:**
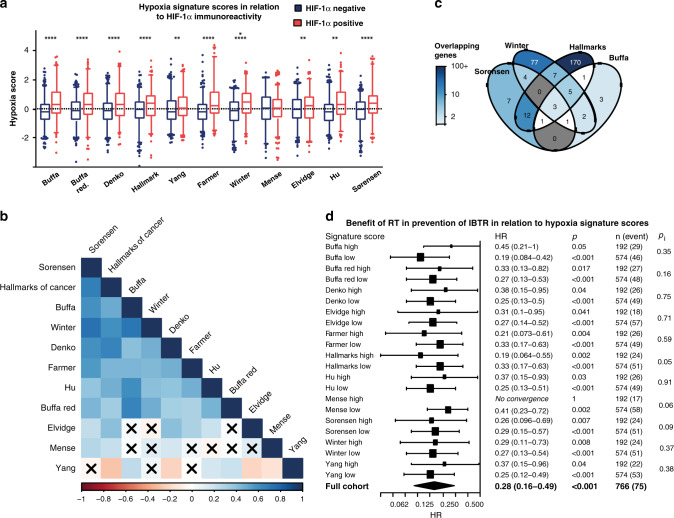
Hypoxic gene-expression signatures. Hypoxia signature scores in relation to HIF-1α IHC status, negative (blue) and positive (red), presented as boxes of the 25th–75th percentiles with whiskers at percentile 2.5–97.5 *p < 0.05, **p < 0.01, ***p < 0.001, ****p < 0.0001 (**a**). Correlations were tested using Spearman’s correlation. Correlation plot of hypoxia signature scores, ordered according to the first principal component order (**b**). Positive correlations in increasing intensity of blue and negative in red. Crossed squares mark correlations where *p* < 0.0001. Venn diagram of gene overlaps in four of the hypoxia signatures between which the scores exhibited the highest correlation (**c**), darker colour representing higher percentage of overlapping genes. Forest plot presenting the benefit of RT in prevention of IBTR the first 5 years after the primary tumour in relation to hypoxia signature scores (**d**). The tumours were stratified based on hypoxia scores, where the 4th quartile was defined as high, and quartile 1–3 as low.

There was a concordance between HIF-1α IHC signal and *HIF-1*α mRNA-expression levels (rho = 0.40, *p* < 0.0001, Fig. 4c). However, there was no association between high *HIF-1*α mRNA expression (highest quartile) and breast cancer recurrence or survival (data not shown). In general, the hypoxic signature scores exhibited a high positive correlation with HIF-1α positive IHC status, with the Mense hypoxia score being the only exception (Fig. 5a). The strongest correlations to HIF-1α positive IHC were observed for “Buffa” (rho = 0.26, *p* < 0.0001), “Farmer” (rho = 0.27, *p* < 0.0001), and “Hu” (rho = 0.26, *p* < 0.0001, Fig. 5a). The calculated hypoxia scores of most gene signatures correlated strongly (Fig. 5b). The Mense hypoxia score did not correlate with the majority of other hypoxia scores, whereas the Yang signature had a clear negative correlation to several other hypoxia scores. Although most hypoxia scores correlated strongly, the gene overlaps were modest with a relatively low number of shared genes (Fig. 5c). Nine genes were present in ≥5 signatures: *ADM*, *NDRG1*, *SLC2A1*, *VEGFA*, *ALDOA*, *IGFBP3*, *LDHA*, *P4HA1* and *TPI1*.

To address the hypothesis that benefit of RT is affected by a hypoxic tumour microenvironment, we evaluated the benefit of RT in relation to gene expression of the hypoxic gene-expression signatures. The cohort was stratified based on the scores of each hypoxia signature and tested for statistical interaction between benefit of RT and hypoxia scores with respect to outcome (Fig. 5d). Patients had benefit from RT in prevention of early IBTR, regardless of hypoxia scores (Fig. 5d).

**Fig. 6:**
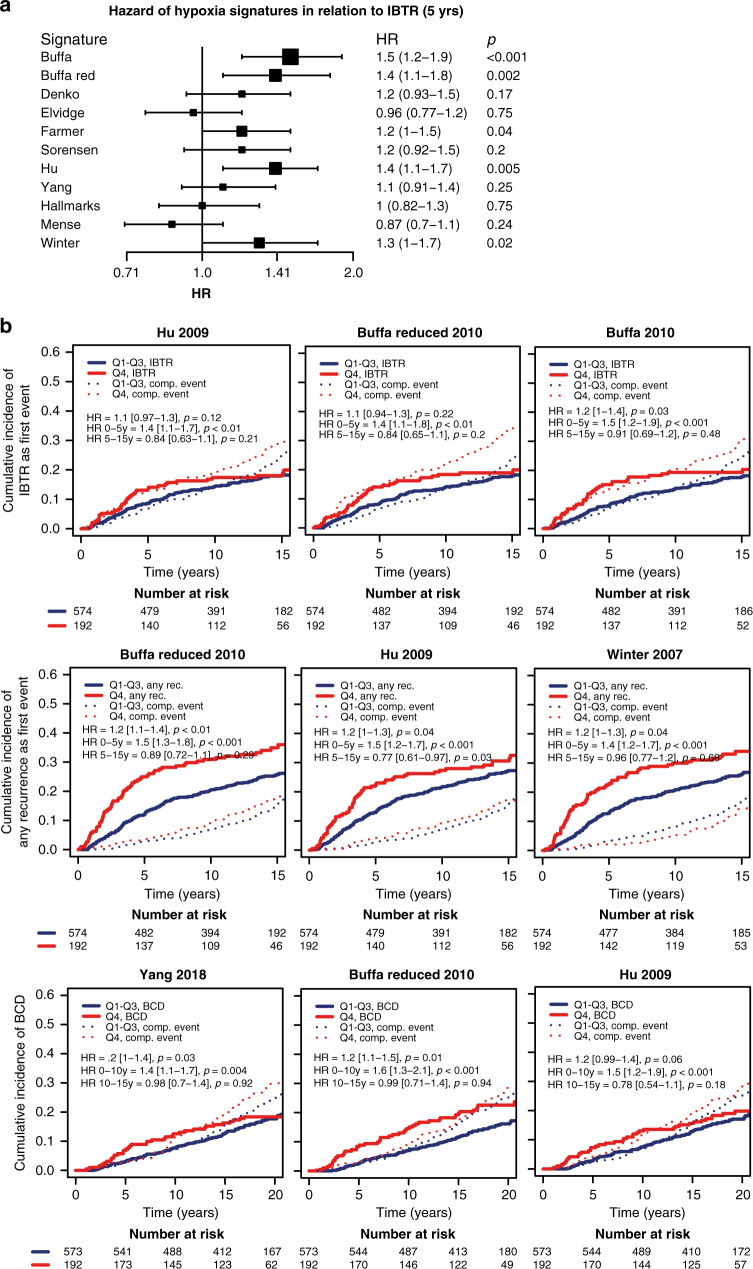
Hypoxic gene-expression signatures in relation to recurrence and BCD. Forest plot presenting HR of hypoxic signature scores as continuous variables in relation to IBTR during the first 5 years after the primary tumour (**a**). For all rows, *n* and events were 766 and 75, respectively. Competing risk curves presenting the relationship between risk of IBTR, any recurrence, or BCD as first event. For each endpoint, the three hypoxia signatures with the strongest association to outcome are shown (**b**). The scores were plotted as a dichotomous variable of high vs. low, where low included quartile 1–3 (Q1–3, blue line), and high included quartile 4 (Q4, red line). Survival data presented as text in the plot area were obtained from Cox proportional hazards model with the score as a continuous variable.

Several signature scores associated with an increased incidence of IBTR as a primary event, with the Buffa signatures (“Buffa” HR_5yrs_ 1.5 [1.2–1.9], *p* < 0.001), and “Buffa reduced” (HR_5yrs_ 1.4 [1.1–1.8], *p* = 0.002) and Hu signature (HR_5yrs_ 1.4 [1.1–1.7], *p* = 0.005) being most prognostic for IBTR (Fig. 6a, b and Supplemental Fig. 3). Additionally, some signatures were associated with any recurrence, with the “Buffa reduced” (HR_0–5yrs_ 1.5 [1.3–1.8], *p* < 0.001), “Hu” (HR_0–5yrs_ 1.5 [1.2–1.7], *p* < 0.001), and “Winter” (HR_0–5yrs_ 1.4 [1.2–1.7], *p* < 0.001) being most pronounced (Fig. 6b and Supplemental Fig. 4). Lastly, some signature scores were associated to an increased incidence of BCD (Fig. 6b and Supplemental Fig. 5), where the “Buffa reduced” (HR_0–10yrs_ 1.6 [1.3–2.1], *p* < 0.001), “Yang” (HR_0–10yrs_ 1.4 [1.1–1.7], *p* = 0.004) and “Hu” (HR_0–10yrs_ 1.5 [1.2–1.9], *p* < 0.001) were most prominent.

## Discussion

In this study, we address the role of tumour hypoxia, detected by HIF-1α IHC or hypoxic gene-expression signatures, in relation to outcome and benefit of RT in a large, randomised trial of postoperative RT in early breast cancer.

In contrast to our initial hypothesis, we show that breast cancer patients benefit from postoperative RT regarding IBTR, and any recurrence, also when the primary tumour was HIF-1α IHC positive or had high expression of hypoxic gene signatures. We found no effect of postoperative RT on BCD, irrespective of primary tumour HIF-1α status. However, a benefit of postoperative RT in the prevention of BCD has been demonstrated in meta-analyses [3]. Thus, RT directed to the remaining breast tissue was similarly effective on disseminated cancer cells whether these were schooled in a hypoxic or non-hypoxic primary tumour.

For breast cancer, RT is most often given after tumour resection. In cancers that are primarily treated with radiation (e.g. some head and neck cancers and bladder cancers) the hypoxic microenvironment remains and may even be enhanced due to radiation-induced tissue damage. The cells residing in the hypoxic microenvironment may then continue communicating with the microenvironment through the release of growth factors and other signalling molecules in a hypoxia-adapted state, potentially affecting the outcome. This could explain why hypoxia has been associated with RT resistance [44–46], but not in this postoperative study.

Tumour HIF-1α positivity correlated to unfavourable tumour characteristics. Analyses of the non-irradiated patient group, as well as the total study population, showed that patients with HIF-1α positive primary tumours were more prone to develop IBTR during the first five years after surgery. Multivariable analysis showed that primary tumour HIF-1α positivity was an independent risk factor for IBTR in the total patient population. In addition, primary tumour HIF-1α positivity correlated to an increase in any recurrence in the whole study population and remained an independent risk factor after adjustment for patient age, tumour subtype, size, and systemic treatment. Primary tumour HIF-1α positivity was also associated with an increase in BCD, but not when adjusted for patient age, tumour subtype, size and systemic treatment. The association between primary tumour HIF-1α positivity and IBTR is, to our knowledge, a novel finding, and in line with previous reports that hypoxia and HIF-1α are associated with distant metastasis and poor prognosis in breast cancer [16–18]. Breast tumour hypoxia and HIF-1α-regulated gene expression contribute to aggressive tumour behaviour and seeding of cancer cells with a metastatic capacity [47, 48].

We found that most tumour samples from surgically removed IBTRs had the same HIF-1α status as their corresponding primary tumour, indicating that hypoxia and HIF-1α positivity is an inherent tumour trait. Additionally, of 11 hypoxia-related gene-expression signatures from the literature, 10 correlated with HIF-1α protein level, and 5 with an increased risk of IBTR within 5 years after resection of the primary tumour. The presence of a HIF-1α-positive IBTR correlated to an increased risk for BCD compared to having a HIF-1α negative IBTR, analysed in relation to time after IBTR-surgery (Fig. 4b).

To specifically address whether ER affects the role of tumour hypoxia, we investigated the hazard of the investigated hypoxia markers in ER-positive and -negative groups separately. We found that primary tumour HIF-1α positivity was a risk factor for IBTR and any recurrence within 5 years after surgery in the ER-positive subgroup as well as in the entire study population. The study included too few patients with ER-negative primary tumour to allow for meaningful multivariable analyses in this subgroup. In this large breast cancer material, we establish that HIF-1α positivity is associated with the tumour subtype. Luminal A tumours have the lowest frequency of HIF-1α positivity, and the frequency of HIF-1α-positivity is then increasing step by step in luminal B, HER2-positive and triple-negative tumours. HIF-1α positivity, thus, has a negative correlation to the distribution of ER expression in the breast cancer subtypes. The ER-negative breast tumours generally have a worse prognosis and a high frequency of HIF-1α-positivity. However, as stated above, within the ER-positive group HIF-1α-positivity remains associated with a worse prognosis. We have previously shown, on the molecular level, that in hypoxic breast cancer cells ER-expression diminishes as HIF-1α accumulate [17, 49]. It is plausible that, in the generally less proliferative ER-positive tumours, the additive effect of growth factors, cytokines and other effectors induced by HIF-regulated transcription have a relatively greater impact than in ER-negative tumours.

Regulation of HIF-1α is mainly post-translational as described above, but we found that HIF-1α IHC signal correlated to *HIF-1α* mRNA-expression levels. Albeit, with a great degree of variability, and *HIF-1α* mRNA-expression did not correlate to tumour characteristics or patient outcome (data not shown). Thus, the need for a hypoxic gene-expression signature remains. To further study the role of tumour hypoxia in relation to patient outcome, we calculated a series of hypoxic scores according to hypoxia-related gene-expression signatures from the literature for each tumour in the cohort. Eleven literature-derived hypoxia gene-expression signatures [30–38] were analysed in relation to patient outcome. There was a high degree of correlation between several of the signatures, which was anticipated as some were published by associated research groups. The signatures were enriched in genes known to be regulated by HIF. Furthermore, we found that their expression correlated with HIF-1α IHC positivity, indicating that IHC detected transcriptionally active HIF-1α. High expression of most of the analysed hypoxia signatures correlated to a higher incidence of IBTR, any recurrence, and BCD, similarly to the pattern seen for HIF-1α IHC positivity. Notably, the Elvidge, Mense and Yang signatures did not correlate to increased occurrence of IBTR and were also among those with the least gene overlap and score correlation with the other signatures (Fig. 5b, c). However, the hallmarks of cancer signature, which had a large overlap with other hypoxic signatures, also did not correlate to increased IBTRs (Fig. 6). None of the hypoxic signatures, similar to HIF-1α IHC, correlated to RT-resistance.

In conclusion, patients with HIF-1α positive primary tumours had a worse outcome with increased recurrences, but these patients still had equal benefit from postoperative RT as patients with a non-hypoxic primary tumour.

## Supplementary information

Supplemental figure 1

Supplemental figure 2

Supplemental figure 3

Supplemental figure 4

Supplemental figure 5

Legends supplemental figures

aj_checklist

## Data Availability

The gene-expression datasets analysed during this study are available at Gene Expression Omnibus with accession number GSE119295. The IHC datasets generated and analysed during the current study are available from the corresponding author on reasonable request.
