# Supplementary material for: Breast cancer hypoxia in relation to prognosis and benefit from radiotherapy after breast-conserving surgery in a large, randomised trial with long-term follow-up
Source: Br J Cancer. 2022 Feb 9;126(8):1145–56. doi: 10.1038/s41416-021-01630-4 (PMC9023448; doi:10.1038/s41416-021-01630-4)
Supplement: Supplementary file 6 — Legends supplemental figures [file 41416_2021_1630_MOESM6_ESM.docx]

**Legends Supplemental Figures**

Supplemental figure 1. Examples of breast cancer TMA cores from the negative (A), low (B), and high (C) HIF-1α IHC staining categories. Size bars 50 μM.

Supplemental figure 2. Cumulative incidence of IBTR (A, B), any recurrences (C, D) as first event, and BCD (E, F) in 985 T1-2N0M0 breast cancer patients randomised to receive RT or not after breast-conserving surgery in patients with HIF-1α negative (blue line) and HIF-1α positive (red line) primary tumour stratified into ER-positive and -negative tumours.

Supplemental figure 3. Forest plot presenting HR of all hypoxic signature scores as continuous variables in relation to IBTR during the first 5 years after the primary tumour.

Supplemental figure 4. Forest plot presenting HR of all hypoxic signature scores as continuous variables in relation to any recurrence during the first 5 years after the primary tumour.

Supplemental figure 5. Forest plot presenting HR of all hypoxic signature scores as continuous variables in relation to BCD during the first 10 years after the primary tumour.
